# The Role and Research Progress of Inhibitor of Differentiation 1 in Atherosclerosis

**DOI:** 10.1089/dna.2021.0745

**Published:** 2022-02-09

**Authors:** Jun Qiu, Youhong Li, BingYu Wang, XinYi Sun, Dingding Qian, Yuchen Ying, Jianqing Zhou

**Affiliations:** ^1^ Department of Cardiology, Medicine School of Ningbo University, Ningbo, China.; ^2^ Department of Cardiology, Lihuili Hospital Affiliated to Ningbo University, Ningbo, China.; ^3^ Department of Cardiology, Ningbo Institute of Innovation for Combined Medicine and Engineering (NIIME), Ningbo, China.

**Keywords:** inhibitor of differentiation, endothelial-mesenchymal transition, angiogenesis, reendothelialization after injury, atherosclerosis

## Abstract

Inhibitor of differentiation 1 has a helix-loop-helix (HLH) structure, belongs to a class of molecules known as the HLH trans-acting factor family, and plays an important role in advancing the cell cycle, promoting cell proliferation and inhibiting cell differentiation. Recent studies have confirmed that inhibitor of differentiation 1 plays an important role in the endothelial-mesenchymal transition of vascular endothelial cells, angiogenesis, reendothelialization after injury, and the formation and rupture of atherosclerotic plaques. An in-depth understanding of the role of inhibitor of differentiation 1 in atherosclerosis will provide new ideas and strategies for the treatment of related diseases.

## Introduction

Inhibitor of differentiation proteins were originally isolated from a mouse red blood cell line in 1990 and belong to the helix-loop-helix (HLH) transcription factor family. These proteins are named Id proteins because they can inhibit the binding of nuclear transcription factors to DNA (Jen *et al*., 1992). It was later discovered that Id proteins are negative regulators of nuclear transcription factors that are widely present in mammalian cells. To date, four Id family members (Id1-4) have been identified, which are encoded by four Id genes (Id1-4) located on different chromosomes. These genes all have a highly conserved HLH homology domain composed of two facultative α-helices (Asirvatham *et al*., 2010). Their main function is to negatively regulate nuclear transcription factors, including suppressing the nuclear transcription of proto-oncogenes.

Atherosclerosis (AS) is the most common cause of cardiovascular disease. Atherosclerosis is driven by the accumulation of intimal lipids and chronic inflammation, and ultimately causes a series of ischemic changes in the organs due to the hardening of the blood vessel wall and the narrowing of the lumen (Falk, 2006). Early endothelial cell damage and repair, inflammation, and angiogenesis in the plaque are involved in the occurrence and development of AS plaques (Jaipersad *et al.*, 2014).

## The Molecular Structure of Id and Its Expression

### Molecular structure of Id

**FIG. 1.**
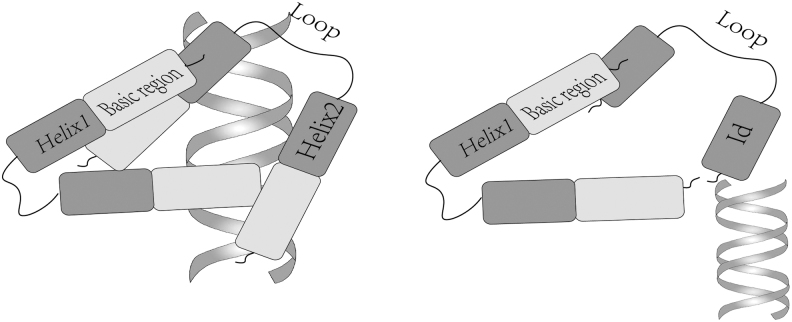
Id1 negatively regulates bHLH proteins and represses target gene transcription. bHLH members (helix1, helix2) recognize specific basic regions of DNA sequences and regulate gene transcription; Id1 protein lacks the basic structural region for DNA binding and cannot bind to DNA. bHLH, basic helix-loop-helix.

The negative transcriptional regulatory factor Id is encoded by four Id genes located on different chromosomes, including 20p11, 2p25, 1p36, and 6p22–21, and forms four subtypes, including Id1, Id2, Id3, and Id4, which belong to the HLH transcription factor family. The HLH is composed of two relatively conserved alpha helices ∼15–20 amino acids in size and a loop region with poorly conserved amino acids between them. Most HLH proteins have basic DNA-binding regions, which can bind to specific DNA sequences to exert biological effects. However, Id lacks similar DNA-binding sites and can bind to basic HLH (bHLH) family members to form a nonfunctional heterodimer, inhibit the transcription of downstream E-box or E-like sequence genes, and exert its dominant negative regulatory effect (Iwanicki and Brugge, 2009) (Fig. 1).

### Id location and expression

The Id gene is an important transcription factor that regulates cell growth and differentiation during biological development. It is widely distributed in various cells and tissues of the human body. Cells and tissues in a resting state express low levels of Id, but in tissues and cells with strong proliferation and differentiation, Id is highly expressed. In a variety of tumor tissues, such as colorectal cancer, prostate cancer, and cervical cancer, Id1 expression is increased and has a certain correlation with tumor progression (Zhao *et al*., 2020).

### Id binding partners

The HLH family is mainly divided into the bHLH and dnHLH (dominant negative HLH) subfamilies. Near the HLH domain in the bHLH subfamily, there is a basic region that can recognize and specifically bind to DNA sequences. This feature is not available in the dnHLH subfamily. Therefore, the dnHLH subfamily cannot bind to DNA, but these proteins perform their biological functions by binding to bHLH subfamily proteins to form a nonfunctional heterodimer, inhibiting the latter's transcriptional activity (Perk *et al*., 2005). The Id protein is a member of the dnHLH subfamily and negatively regulates bHLH proteins. Id mainly targets E proteins (such as E12, E47, E2–2, and HEB). These proteins belong to class A bHLH transcription factors and are expressed in almost all cells (Sawada and Littman, 1993; Peverali *et al.*, 1994). The expression of B-type bHLH transcription factors has tissue specificity, such as the specific expression of SCL in cardiomyocytes (Org *et al.*, 2015). MyoD and myogenin are distributed in muscle tissue (Ganassi *et al.*, 2018). Fat tissue express Myf-5 (Wu *et al.*, 2012).

## Regulation of Id1 Expression by transforming growth factor-β/bone morphogenetic protein

Members of the transforming growth factor-β (TGF-β) family are multifunctional cellular molecules, include TGF/Activin/Nodal and bone morphogenetic protein (BMP)/GDF, and are essential in regulating the growth, development and differentiation of cells and tissues. The TGF-β signaling pathway is activated by ligand binding to TGF-β type II receptors, phosphorylating TGF-β type I receptors (ALK1 and ALK5), and then phosphorylating two R-Smad proteins, namely, Smad2/3 and Smad1/5; activated R-Smad and Smad4 form a complex in the nucleus to regulate target gene expression (Budi *et al*., 2017), such as that of plasminogen activator inhibitor-1 and Id proteins (Id1, Id2, Id3 and Id4). As one of the main members of the TGF-β family, BMP plays an important role in bone development, homeostasis and diseases (Lowery and Vicki, 2018). Id1 is the direct target gene of BMP, and BMP-induced Id gene expression has been shown in different cell lines and embryonic stem cells. BMP upregulates Id1 expression without *de novo* protein synthesis (Korchynskyi and ten Dijke, 2002). The Smad-binding element and GC-rich region in BMP regulate the expression of the Id1 gene by binding to Smad1 and Smad5 (Gao *et al.*, 2021). Interestingly, the vasodilatation observed in Id1 and Id3 double-knockout embryos is similar to the defective blood vessels in BMP-specific Smad1 or Smad5 knockout (Chang *et al.*, 2020), indicating that the Id1 gene may be a key mediator of BMP activation in ECs.

As Id1 is related to the bHLH transcription factor, it is a differentiation inhibitor (Zebedee and Hara, 2001). Therefore, the early induction of Id1 may be the mechanism by which BMP inhibits differentiation by controlling the activity of the bHLH protein. For example, in neurodevelopment, BMP is an important inhibitor of neuronal differentiation, and transient induction of Id1 by BMP may reduce the stability of the neurogenic bHLH transcription factor Mash1 (Viñals *et al.*, 2004). Similarly, the combination of Twist1 and E protein (E47) inhibits BMP signal-mediated differentiation of mesenchymal cells into osteoblasts, and Id1 induced by BMP can relieve this inhibitory effect (Hayashi *et al*., 2007). Therefore, we hypothesize that under the effects of BMP, Id1 induction helps to inhibit the bHLH transcription factor that is involved in the differentiation of a variety of cell lines.

However, the inhibition of cell growth and partial differentiation induced by TGF-β is related to the downregulated expression of the gene encoding the Id protein (Huan *et al.*, 2015). The induction of Id1 by TGF-β is determined by the particular levels in the cells (Li *et al.*, 2007). In many epithelial cell lines, TGF-β downregulates Id1 expression (Di *et al.*, 2006; Cao *et al.*, 2009) and upregulates Id1 expression in mouse hepatic stellate cells and human fetal lung fibroblasts (Chambers *et al.*, 2003) (Wiercinska *et al.*, 2006). In addition, the early induction of the Id1 gene by TGF-β depends on Smad3 but not Smad2. This effect is related to the binding of Smad3 to the upstream region of the Id1 promoter (Liang *et al*., 2009). ECs treated with TGF-β exhibit a biphasic response: at low concentrations, the migration and proliferation of ECs are activated, while at high concentrations, these processes are inhibited (Goumans *et al.*, 2002). This effect depends on the activation of two different TGF-βI receptors, ALK1 and ALK5. Low concentrations of TGF-β activate ALK1 signaling through Smad5, which can upregulate the expression of Id1 and increase the migration and proliferation of ECs. Under higher doses of TGF-β, Alk5 is activated, which induces PAI and may inhibit the migration and proliferation of ECs by downregulating Id1 (CelineSouilhol *et al.*, 2018).

In short, as one of the most specific downstream target genes of TGF-β/BMP, Id1 can not only potentially modulate the cellular effects of BMP signaling but also regulate Id1 to maintain the proper response of cells to TGF-β.

## Id1 Regulation of ECs and Reendothelialization

### Id1 and senescence

Senescence is related to the gradual decline of cardiovascular structure and function. The increase in cardiovascular disease in aging is partly the result of the aging of vascular ECs and related vascular dysfunction. During this process, EC senescence is a pathophysiological process associated with structural and functional changes, including vascular tone disorders, increased endothelial permeability, arterial stiffness, impaired angiogenesis and vascular repair, and decreased EC mitochondrial biogenesis. Cellular senescence manifests as functional declines and low metabolism such as cell cycle arrest (Sabbatinelli *et al.*, 2019).

It has been proposed that EC aging is mainly caused by telomere shortening (Bodnar *et al.*, 1998). The expression of exogenous TERT has been shown to restore telomere length and extend cell lifespan (Duncan *et al*., 2000). Subsequent research showed that growth arrest of cultured human fetal cardiomyocytes was accompanied by an increase in p16INK4A and a decrease in Id gene expression (Ball and Levine, 2005). p16INK4A/pRb is the main mediator of cellular aging. p16INK4A-mediated senescence works through the retinoblastoma (Rb) pathway, inhibiting cyclin-dependent kinase (CDK) and leading to G1 cell cycle arrest (Anerillas *et al.*, 2020). Id1 delays cell senescence mainly by regulating the expression of p16INK4A (Akakura *et al.*, 2010). This finding indicates that the expression of Id1 is sufficient and necessary for the inhibition of p16INK4A. This effect is mediated at least, in part, by the inhibition of DNA binding of Ets2 transcription factors by Id1 (Ohtani *et al.*, 2001). Although it has been suggested that Id inhibition of E protein also mediates the inhibition of p16INK4A, E47 overexpression does not upregulate the expression of endogenous p16 in 293T cells (Zheng *et al.*, 2004). In addition, cells lacking endogenous Id1 have different senescence characteristics than ordinary cells. This phenomenon may be due to Id1 not completely inhibiting the expression of p16INK4A because other members of the Id family may also participate in cell senescence by affecting the p16INK4A/Rb pathway. For example, Id2 can bind to Rb and enhance S phase progression by weakening the growth inhibitory activity of the Rb protein (Strait *et al.*, 2002).

### Id1 and ECs activation

The proliferation and migration of ECs (also known as EC activation) play important roles in angiogenesis, wound healing, and endothelial injury healing. Particularly in certain stages of the pathology of certain cardiovascular diseases, such as atherosclerosis, the proliferation and migration of ECs play extremely important roles (Draoui *et al*., 2017). On the one hand, EC activity can restore the integrity of the endothelium; on the other hand, activated ECs can participate in the formation of new capillaries and participate in damage repair. Id1 is highly expressed in damaged ECs. Dilation, discontinuity, leakage, and apoptosis begin to appear in ECs 1 week after the ablation of Id1, and the severity increased over time, indicating the characteristics of EC aging, eventually leading to the destruction of vascular integrity (Gadomski *et al.*, 2020).

**FIG. 2.**
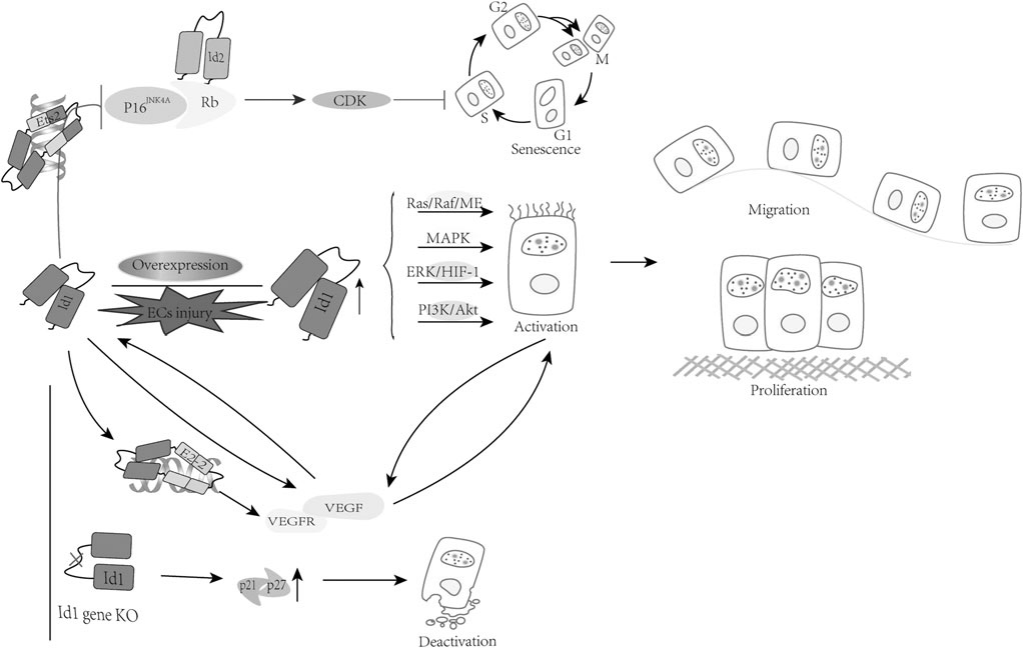
Id1 affects EC senescence, proliferation, and migration. Id1 inhibits DNA binding of the Ets2 transcription factor to suppress p16INK4a expression in turn delaying EC senescence; the effects of Id1 upregulation are mediated through the regulation of Ras/Raf/ME, MAPK, ERK/HIF-1α, PI3K/Akt, while regulating ECs proliferation and migration; the positive feedback loop formed by Id1 with VEGF regulates ECs activation and interacts with E2–2 to relieve e2-2-mediated inhibition of VEGFR2 expression. Id1 knockdown leads to increased p21 and p27 expression and ECs are deactivation. CDK, cyclin-dependent protein kinase; EC, endothelial cell; ERK/HIF-1, extracellular signal-regulated kinase/hypoxia inducible factor-1; MAPK, mitogen-activated protein kinase; PI3K/AKT, phosphoinosmde-3-kinase/protein kinase B; Rb, retinoblastoma; VEGF, vascular endothelial growth factor; VEGFR, vascular endothelial growth factor receptor.

BMP/Smad activation of EC migration and tube formation depends on the activation of Id1. This process may be caused by changes in the activity of transcription factors, leading to changes in the expression of metalloprotease and integrin genes (Valdimarsdottir *et al.*, 2002). Overexpression of Id1 can also promote EC migration and tube formation, thereby simulating the effect of BMP/Smad on ECs. The upregulation of Id1 caused by endothelial injury can regulate the activation of ECs in a variety of ways (Fig. 2). Id1 can directly upregulate the expression of vascular endothelial growth factor (VEGF) and promote the proliferation and migration of ECs. The proliferation and migration of ECs can also promote the transcription of VEGF, which in turn promotes the expression of Id1, forming a positive feedback loop (Scharpfenecker *et al.*, 2007). Another possible mechanism by which Id1 enhances ECs activation is as follows: by inhibiting bHLH transcription factor activity, E2–2 inhibits ECs proliferation, migration, and network formation by inhibiting vascular endothelial growth factor receptor 2 (VEGFR2) promoter activity, and this effect can be antagonized by Id1 (Tanaka *et al.*, 2010). Id1^−/−^ ECs showed increased expression of the CDK inhibitors p21 and p27 and impaired proliferation, which could be reversed by reducing the expression of E2–2 (Gadomski *et al.*, 2020).

### Id1 and reendothelialization

Endothelial injury is the main cause of AS and other cardiovascular diseases. When blood vessels are injured, endothelial progenitor cells (EPCs) are mobilized from the bone marrow into the blood, proliferate and migrate to the vascular injury site, and differentiate into mature ECs to facilitate vascular repair and endothelialization (Del Papa and Pignataro, 2018). In recent years, the potential clinical significance of EPCs has received increasing attention due to their key role in reendothelialization. The proliferation and migration of EPCs is the key mechanism of reendothelialization after vascular injury. Past studies have shown that Id1 can be used as a marker of EPCs to track EPCs in bone marrow, blood, and tumor stroma. Id1 gene silencing can ablate bone marrow-derived EPCs and cause obvious defects in angiogenesis-mediated tumor growth (Moschetta *et al.*, 2016). In addition, the production of EPCs in the bone marrow relies on Id1 inhibition of its target gene p21 (Ciarrocchi *et al.*, 2007). The Id1 protein regulates the proliferation and migration of EPCs through various mechanisms and regulates the process of endothelialization (Wang *et al.*, 2010). Id1 interacts with E2–2 to release E2-2-mediated FGFR1 and VEGFR2 expression inhibition to regulate EPC function (Yu *et al.*, 2016). In addition, Id1 regulates the cell cycle to promote the proliferation of EPCs and induces the release of paracrine signals derived from EPCs to enhance the viability of neighboring ECs. These effects depend on the activation of Wnt2 signals (Xia *et al.*, 2016). Percutaneous coronary intervention (PCI) is the most commonly used procedure for treating atherosclerosis and benefits most patients with AS, but there is still the risk of mechanical damage to the vascular endothelium, which will eventually lead to restenosis of the target vessel. Li *et al.* used a balloon injury rat model to simulate patients after PCI and found that Id1 could inhibit early reactive intimal hyperplasia and promote the reendothelialization of injured blood vessels through NF-κB/survivin signal transduction (Li *et al*., 2019b). Mechanistically, Id1 affects the mobilization and proliferation of EPCs and promotes angiogenesis through NF-κB signaling. Id1 gene transfer endows human umbilical vein endothelial cells with angiogenic properties and rescues mice with ischemic limbs (Nishiyama *et al.*, 2005).

## Id1 and EMT/Endothelial–Mesenchymal Transition

Vascular endothelial cells (VECs) are a continuous physical barrier between the blood and the inside of the vessel wall that can maintain the stability of the vascular environment (Bischoff, 2019). Endothelial dysfunction usually refers to impaired endothelium-dependent vasodilation and increased contraction. This change is considered an early event of AS (Hong *et al.*, 2018). Therefore, maintaining vascular endothelial function and preventing VECs damage is an important direction in the AS research field. VECs undergo endothelial–mesenchymal transition (EndMT) when stimulated by blood flow shear force, inflammatory factors, high fat, and other factors, which damages the barrier function of the endothelium (Sanchez-Duffhues *et al*., 2016). EndMT is a state of phenotypic transformation from ECs to mesenchymal cells in which ECs-specific markers (VE-cadherin, PECAM-1) are downregulated and mesenchymal cell-specific markers (α-SMA, Fibronectin, vimentin) are upregulated, with characteristic cell morphology and changes in proliferation, migration, and collagen synthesis ability (Nieto *et al.*, 2016; Li *et al*., 2018). Recent studies have shown that EndMT plays an important role in heart and kidney fibrosis, pulmonary hypertension, cancer, and other diseases (Grande *et al.*, 2015), and research on its role in the process of AS is also intensified. Chen *et al.* (Evrard *et al.*, 2016) used endothelial-specific lineage tracking and found that there were EndMT-derived ECs in AS plaques. These cells were related to the unstable plaque phenotype. Most studies have shown that EndMT is related to a reduction in Id protein expression (Moonen *et al.*, 2010). Id1 promotes tumor metastasis and colonization by activating the EMT program in tumor cells (Castañón *et al.*, 2017). Id1 induces the expression of mesenchymal markers (vimentin and fibronectin) by downregulating the zinc finger inhibitor protein ZBP-89 (Pillai *et al.*, 2011).

**FIG. 3.**
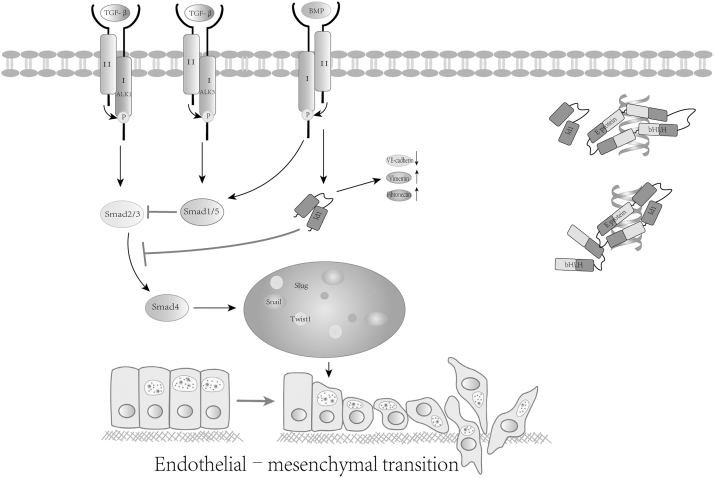
Id1 regulates the EndMT process. TGF-β and BMP signaling through the type II receptor activates the type I receptor, which activates Smad2/3 and Smad1/5, which form a complex with Smad4 into the nucleus to regulate EndMT-related transcription factor (Snail, Twist, Slug) expression and affect EndMT process. Id1 antagonizes Smad2/3 signaling and affects snail, twist, and Slug expression. Id1 decreased endothelial marker VE-cadherin and upregulated mesenchymal markers vimentin, fibronectin levels. Id1 competitively inhibits the binding of bHLH to the E-box in the promoter of the VE-cadherin gene, blocking the transcription of the VE-cadherin gene. BMP, bone morphogenetic protein; EndMT, endothelial–mesenchymal transition; TGF-β, transforming growth factor-β.

Id1, as the most specific downstream target gene of Smad1/5, is important in antagonizing Snail (Castañón *et al.*, 2017), Twist1 (Stankic *et al.*, 2013a), and Slug (Asirvatham *et al*., 2007), which are induced by Smad2/3. TGF-β can also activate non-Smad pathways, such as ERK/MAPK, which regulates Id1 expression at the transcriptional level to facilitate the cross talk between non-Smad pathways and Smad pathways, regulate Smad2/3 phosphorylation, and ultimately regulate the EndMT process (Medici *et al*., 2011) (Fig. 3).

In addition, BMP, a member of the TGF-β family, promotes the migration of ECs and the formation of cardiac ducts by inducing the expression of Id1 (Jin *et al.*, 2018). BMPs inhibit the EndMT process by activating Smad1/5 (Zhang *et al.*, 2020). The BMPR-deficient ECs phenotype shifts to a contractile myofibroblast phenotype during actin bundle and myosin contraction, indicating that these cells undergo EndMT (Hiepen *et al.*, 2019). LDH-198139 can inhibit BMP-Smad1/5 pathway signaling and reduce the level of Id1 protein. LDH-198139 was injected into Matrix Gla protein-null (MGP^−/−^) mice and was shown to prevent EndMT, cause a reduction in vascular calcification, and increase the survival rate in MGP^−/−^ mice (Malhotra *et al.*, 2015). In addition to the classic pathway, Wnt/β-catenin has also been reported to be involved in the regulation of EndMT (Li *et al.*, 2019a). Id1 is a downstream target gene. Id1 knockout reduces the expression of β-catenin in the cytoplasm/nucleus and T cell factor/lymphoid enhancer factor luciferase activity and reduces expression of the Wnt target genes cyclinD1 and Survivin. Therefore, Id1 inhibits Wnt/β-catenin signaling and the EMT process (Sun *et al.*, 2019).

In some systems, the role of Id1 in EMT seems to depend on its dominant negative effect on the E protein. Id1 binds with HEB to form a heterodimer and inhibits the binding of HEB and E-Boxes in the VE-cadherin gene promoter, thereby blocking the transcription of the VE-cadherin gene (Li *et al.*, 2007). Cubillo *et al.* (2013) used chromatin immunoprecipitation to show that in complexes lacking Id1, E47 can directly bind to the endogenous VE-cadherin promoter in EndMT cells. In other cases, Id1 mediates the EMT process by interacting with cytoplasmic/membrane proteins (Zhang *et al.*, 2007).

Studies have shown that the expression of Id1 is necessary to maintain the mesenchymal phenotype and cell viability after EndMT (Cubillo *et al.*, 2013). The maintenance of EndMT status at the tumor metastatic colonization site depends on the Id1 target protein Twist1, while the maintenance of this cell status at the original site depends on Snail1 (Stankic *et al.*, 2013b). Both Twist1 and Snail can drive EndMT to promote the development of atherosclerosis (Mahmoud *et al.*, 2017). Id1-knockout colon cancer EMT cells showed increased VE-cadherin and decreased Snail and Twist, indicating that regulating Id1 may have good prospects for reversing EndMT. It is worth noting that in the early stage of TGF-β1-mediated EMT, Id1 can only inhibit the expression of ECs markers (VE-cadherin and ZO-1), but markers representing the mesenchymal phenotype (α-SMA, matrix metalloproteinase [MMP]-2, fibronectin, and integrin-linked kinase) did not change significantly (Li *et al.*, 2007). This finding indicates that Id1 cannot induce complete EMT, which indirectly indicates that EMT is a multistep process, in which the loss of epithelial adhesion does not necessarily lead to complete mesenchymal transition.

## Id1 and Angiogenesis

There is increasing evidence that angiogenesis is a key factor in the growth and instability of AS plaques (Parma *et al.*, 2017). During the development stage of AS, a variety of inflammatory stresses, oxidized lipids, and proteases stimulate the preexisting vasa vasorum in the plaque to generate new blood vessels, but the structure of these new blood vessels is immature and lacks the protection of the elastic layer and smooth muscle cells. Cytotoxic substances (such as oxidized lipids and oxidative stress) are produced in the plaque, causing damage to the plaque, leading to plaque rupture, and ultimately leading to death (Camaré *et al.*, 2017). Therefore, inhibiting neovascularization in the plaque is a potential therapeutic strategy for atherosclerosis.

Angiogenesis is a complex process that includes cell proliferation, migration, basement membrane degradation, and the maturation of new blood vessels (Xu *et al.*, 2021). Early studies showed that in mouse models of cancer, in neovascularization, Id1 was necessary for normal blood vessel formation at the primary and metastatic sites of tumors (Gao *et al*., 2008). The blood vessels in Id1-KO animals lack the ability to branch and germinate and cannot form normal caliber lumens (Benezra *et al.*, 2001). In ECs, Id1 can be upregulated by VECs growth factor (VEGF-A). VEGF-A is one of the most important factors that stimulates atherosclerotic plaque rupture (Wang *et al.*, 2011). Id1 can also activate VEGF and promote angiogenesis through an autocrine pathway (Ling *et al.*, 2005). The two factors interact to promote EPCs and mobilize hematopoietic precursors in the bone marrow. At present, Id1 is also considered to be a proangiogenic factor that is closely related to angiogenesis, in addition to VEGF, which is very important in AS-associated angiogenesis. MMPs can degrade extracellular matrix, destroy basement membrane, and affect angiogenesis in AS plaques (Chow *et al*., 2007). Id1 regulates the expression of MMP-14 and MMP-17, affects collagen degradation, and promotes ECs migration and angiogenesis in plaques (Chen *et al.*, 2017).

A large number of studies have shown that different concentrations of ox-LDL play important roles in AS angiogenesis (Singh and Gautam, 2019; Liu *et al.*, 2020). Low concentrations (<20 μg/mL) of ox-LDL induce Id1 nucleocytoplasmic shuttling through the PI3K pathway to promote ECs angiogenesis, thereby promoting plaque vulnerability and intravascular thrombosis (Qiu *et al.*, 2012). High concentrations (>20 μg/mL) of ox-LDL reduce the proliferation and migration of ECs, and the level of nitric oxide accelerates AS. Id1 overexpression eliminates this inhibitory effect, which is achieved by inhibiting the nuclear translocation of P53 (Qiu *et al.*, 2011b). In the early days, P53 was considered to be a tumor suppressor. Many studies have shown that an increase in p53 greatly promotes the development of CVD in chronic cardiovascular diseases through antiangiogenesis, programmed cell death, regulation of metabolism, and cell cycle arrest (Men *et al*., 2021). Qiu *et al.* (Wang Guixue *et al.*, 2011) established a rabbit carotid artery stenosis model and found that vulnerable plaques mainly occur in the high shear stress area near the heart in the stenosis vessel. This area mainly promotes the formation of vulnerable plaques by promoting immature angiogenesis in the plaques. The Id1–p53 signaling pathway is an important signaling pathway through which shear stress regulates angiogenesis. Id1 and p53 are closely related to the regulation of angiogenesis. Id1 enhances migration and tubule formation by controlling the expression and function of p53 (Lee *et al.*, 2009; Qiu *et al.*, 2011a). In turn, p53 inhibits Id1 activity through its target DEC1 (Qian and Chen, 2008). In ECs, fluvastatin mediates the proangiogenic effects of statins by regulating Id1 and P53 (Pammer *et al.*, 2004).

Interestingly, low-grade inflammation can also inhibit angiogenesis. For example, TNF-α, a classic inflammatory molecule, can transmit antiangiogenic signals through P53 to inhibit the activity of the Id1 protein (Panta *et al.*, 2017). Trru *et al.* (2008) used TaqMan low-density arrays and showed that C-reactive protein could indirectly induce the protein expression of Id1 and regulate neovascularization in the inner membrane of vulnerable plaques. Recently, drugs that antagonize Id1 have shown preliminary promise in the treatment of pathological fundus angiogenesis (Wojnarowicz *et al.*, 2019). Therefore, inhibiting Id1 is a good antiangiogenic strategy and may play an important role in the pathological process of atherosclerotic plaque instability.

## Id1 and Atherosclerotic Plaque

Multiple studies have shown that Id1 is regulated by shear stress and may be an important force-sensitive factor. Zhang *et al.* (2018) constructed a low oscillation shear stress (OSS) model by ligating the carotid artery in ApoE^−/−^ mice. Different shear stresses have different effects on Id1. Low shear stress (LSS 5 dyn/cm^2^) induced the continuous expression of Id1, but the effect of low oscillating shear stress (OSS 0.5 + 4 dyn/cm^2^) on Id1 was affected by time and was finally inhibited after a short activation. After Ni *et al.* (2010) performed gene microarray analysis, the expression of the Id1 gene in VECs was shown to be regulated by shear stress. However, there has been no further research report on the changes in endothelial function caused by the expression of Id1 under shear stress. In future studies, it will be interesting to determine whether shear stress-induced Id1 changes the functional state of ECs and contributes to subsequent plaque inflammation and instability.

## Conclusions

In summary, as a dominant negative regulator, Id1 plays an important role not only in cell growth, development, cell cycle regulation, and tumor generation but also in EndMT, angiogenesis, re-endothelialization after injury, inflammation, and atherosclerosis. Id1 plays an important role in the occurrence and prevention of atherosclerotic plaque and provides a new target for the prevention and treatment of atherosclerosis in the future. However, the current role of Id1 in cardiovascular disease and its mechanism are not yet well understood, and there are still many aspects, such as its role in the formation and development of coronary heart disease and prevention and treatment, that are still unclear, and more research and discussion are needed.
